# Realizing Impact of Artificial Intelligence in Real World Enhances Public Health

**DOI:** 10.3389/ebm.2025.10700

**Published:** 2025-06-27

**Authors:** Huixiao Hong, William Slikker

**Affiliations:** ^1^ Division of Bioinformatics and Biostatistics, National Center for Toxicological Research, US Food and Drug Administration, Jefferson, AR, United States; ^2^ Retired, Formerly from the National Center for Toxicological Research, US Food and Drug Administration, Jefferson, AR, United States

**Keywords:** AI, bioinformatics, machine learning, deep learning, data

This thematic issue is a product of the 10th annual conference of the Arkansas Bioinformatics Consortium (AR-BIC), which was held on February 26–27, 2024, in Little Rock, Arkansas, with a theme “Real World Impact of AI”. This conference gathered more than 200 scientists and trainees with diverse scientific interests discussing current research works and future perspectives on realizing the impact of artificial intelligence (AI) in the real world. The conference hosted three pre-conference workshops that provided the attendees with state-of-art knowledge and tools on real world applications of AI, including imaging and genomic data analysis. In the plenary presentations, distinguished scientists gave perspectives on how AI transforms toxicology, drug discovery, and public health, calling attentions to both emerging opportunities and practical applications. Four breakout sessions covered presentations from front-line experts to share their cutting-edge research on application of AI to various vital fields, such as natural language processing (NLP) for pharmacovigilance, ethical frameworks for responsible AI, and novel models in spatial omics and computational toxicology. Of special note is the high-profile dialogue with former US FDA chief scientist Dr. Namandjé Bumpus and the NIEHS/NTP director Dr. Richard Woychik on applications of AI in regulatory sciences. The conference exhibited real world impacts of AI, focusing on transformative roles of AI in bioinformatics and public health. The papers included in this thematic issue are from participants of this conference and demonstrate the scientific efforts of AR-BIC to realize the impact of AI in the real world.

Compounds interacting with the endocrine system can lead to numerous adverse outcomes. The androgen receptor is one important component in the endocrine system and mediates male sex hormones. Chemicals binding to androgen receptor raise concerns on reproductive health. Therefore, a high-quality data source of androgenic activity data is urgently needed to fully utilize artificial intelligence techniques such as machine learning and deep learning to develop reliable models for predicting androgenic activity of compounds. [1] introduced the Molecules with Androgenic Activity Resource (MAAR) that was developed to facilitate utilization of androgenic activity data for assessing chemical risk. MAAR is an open-access data source designed to provide comprehensive data for developing machine learning and deep learning models and streamlining and improving the evaluation of androgenic activity of compounds. This tool has a user-friendly interface, providing for efficient navigation and download of the androgenic activity data. The open-access nature of MAAR facilitates the use of androgenic activity data in the development of machine learning and deep learning models for assessing chemical risk, supporting regulatory reviewers and scientists in evaluating the endocrine-disrupting potential of compounds.

Pharmacogenomics plays a vital role in precision medicine. However, the available genomic data of drugs are distributed in diverse data sources, making access to the pharmacogenomics data time-consuming and thus hindering the implementation of precision medicine. Therefore, tools are needed to enable rapid and automatic identification of sources that contain high-quality pharmacogenomics data. [2] explored the ability of large language models in this role. They tested the feasibility of Llama3.1–70B in extracting pharmacogenomics data from the FDA Table of Pharmacogenomic Biomarkers in Drug Labeling (https://www.fda.gov/drugs/science-and-research-drugs/table-pharmacogenomic-biomarkers-drug-labeling) as an alternative approach to the most used labor-intensive methods. The results showed a high accuracy in identifying genomic biomarkers of drugs from single labeling texts or mixed texts, demonstrating the effectiveness Llama3.1–70B in analyzing pharmacogenomics data. This study showcases the applicability of large language models to extract pharmacogenomics data from unstructured scientific and regulatory documents, paving the way for promoting precision medicine.

Screening tests for disease is important for improving diagnosis reliability. Performance of disease screening tests are typically measured using metrics such as sensitivity, specificity, and positive predictive value, quantifying the goodness of tests in differentiating between those with and without a disease. It is well known that these performance metrics, especially positive predictive value, are not reliable for traditional screening tests when the prevalence is very low. Machine learning algorithms are gaining popularity in developing predictive models to serve as *in silico* screening tests for disease. However, the screening and diagnostic performance of *in silico* screening tests, particularly for low prevalence cohorts, has not been fully investigated. [3] used The Strong Heart Study (https://strongheartstudy.org/), a study of cardiovascular disease and its risk factors among American Indians, as a case study to evaluate screening test diagnostics of *in silico* models, built with machine learning algorithms logistic regression, artificial neural networks, and random forest, at varying prevalence. Their results revealed that although sensitivity was not greatly affected in these *in silico* screening tests, specificity and positive predictive values dramatically declined when the prevalence decreased. This study demonstrates that machine learning models as disease screening tests have the same limitations as traditional screening tests when the disease prevalence is low in the testing cohort, calling for further studies to explore reliable *in silico* models for disease screening of low prevalence cohorts.

Natural language processing is an artificial intelligence branch and plays an important role in pharmacovigilance studies. Traditional topic modeling, such as Latent Dirichlet Allocation (LDA), has been widely used in text mining. However, LDA has limitations in capturing the semantic relationships in textual data, which is crucial in natural language processing. Bidirectional encoder representations from transformers (BERT) model-based topic modeling, BERTopic, can capture the contextual relationships. [4] integrated artificial intelligence modules to LDA and BERTopic and compared the two methods in evaluating prescription opioid-related cardiovascular risks in women by analyzing PubMed abstracts. Their results showed that that artificial intelligence algorithms can improve the performance of both LDA and BERTopic in identifying adverse events associated with prescription opioid drugs. Their comparison indicated while LDA remains useful for analyzing large-scale text at low computational cost, BERTopic can enhance interpretability and improve semantic coherence for extracting information in textual data.

Opioids are powerful pain-relieving drugs that are widely used in clinical practice. However, opioid addiction is a serious concern and can lead to opioid use disorder. Opioid drugs bind to opioid receptors, including the μ opioid receptor (MOR), attaining analgesic effects. Therefore, to develop pain treatment drugs that binding opioid receptors but are less addictive is one of the approaches to combat the opioid crisis. With the advancement of artificial intelligence and availability of experimental data, machine learning and deep learning have gained interest in new drug development. [5] developed models for predicting MOR binding activity of compounds using various machine learning and deep learning algorithms for assisting the development of less addictive drugs that target MOR. Their models have been assessed using both internal and external validations and have demonstrated robust predictive performance. The results suggest that the developed models could be used to predict MOR binders, potentially assisting in the development of less addictive drugs. This study demonstrates that machine learning and deep learning models can be used to guide the design of less addictive analgesics and ultimately lead to enhanced patient health.

Unstructured data such as textual documents in scientific publications, social media platforms, and clinical reports are often used for drug safety surveillance. One of the tasks in pharmacovigilance studies is to identify adverse events associated with drugs. Usually, different names can be used for the same drug in textual documents, making it challenging to determining drugs associated with the identified adverse events in drug safety surveillance. Therefore, a comprehensive, non-redundant, and accurate list of drug names is crucial for identification and analysis of adverse events associated with drugs. RxNorm stands out from many sources of drug names as the most popular source used in pharmacovigilance studies. However, the effectiveness of drug names in RxNorm for drug safety surveillance needs to be thoroughly assessed. [6] examined the drug names in RxNorm and developed a refined set of drug names for enhancing unstructured data analysis in drug safety surveillance. They removed duplicates, false drug names, and drug names likely causing inaccurate drug counts in drug safety surveillance from RxNorm, yielding a refined set of drug names. The efficiency and accuracy of the refined drug names were evaluated and compared with the names of original RxNorm using PubMed abstracts. The results demonstrated an increased computational efficiency and decreased false drug names identified for the refined set. Their findings indicate that the refined drug names can improve identification and counting of drugs in unstructured textual data, thereby improving pharmacovigilance.

Mass cytometry is widely used for high-throughput characterization of cellular heterogeneity. Analyzing experimental data from mass cytometry often employ manual gating or clustering technique. [7] proposed quantized optimal transport (QOT), a novel framework derived from optimal transport theory, to analyze mass cytometry data. They used QOT to measure distances between samples based on cellular protein expression profiles by treating the cell-by-protein matrix as a high-dimensional distribution. Their method enables a direct distribution comparison to capture small variations in mass cytometry data and does not need predefined gating strategies. This method was evaluated using two time-series mass cytometry datasets of Coronavirus Disease 2019 (COVID-19) samples. Their leave-one-out analysis identified CD3 and CD45 as immunologically unstable proteins which had the most variation over time during the vaccine response. Their hierarchical clustering based on pairwise Wasserstein distances between samples resulted in the discovery of optimal combinations of immunological markers for grouping samples of different time points from the same patients. This study demonstrates that QOT is a reliable and flexible method for analysis of mass cytometry data of patients to capture immune response heterogeneity, improving the identification of unstable immunological markers and improving patient health.

Antiretroviral therapy (ART) is effective for mitigating human immunodeficiency virus transmission from mother to child. However, there are concerns on potential long-term impacts of ART on offspring health. The gut microbiome contains a huge number of microorganisms, including many types of bacteria. The population of gut bacterial and the produced short-chain fatty acids in the offspring of an ART treated mother can be used to evaluate the health effects of the offspring. [8] investigated the potential long-term effects of ART on offspring health through analyzing gut microbiota populations and short-chain fatty acids concentrations in aged rat offspring with ART exposure *in utero* and during lactation. In this study, pregnant rats received a combination of antiretroviral drugs at two different doses during gestation and lactation, and their offspring’s fecal bacterial abundance and short-chain fatty acid concentrations at 12 months of age were analyzed. They found that *Firmicutes* in males were decreased, while *Actinobacteria* in both males and females were increased. However, the metabolic products (short chain fatty acids) and immune factors (IgA) remained stable. This study suggests a need for further understanding of the long-term effects of ART on offspring and points to future pathways for monitoring offspring health.

Collectively, these articles highlight the advances accomplished to demonstrate the impact of AI in the real world, both to revolutionize biomedical research and enhance public health.

This editorial reflects the views of the authors and does not necessarily reflect those of the U.S. Food and Drug Administration.
